# Efficacy of preemptive analgesia versus postoperative analgesia of celecoxib on postoperative pain, patients’ global assessment and hip function recovery in femoroacetabular impingement patients underwent hip arthroscopy surgery

**DOI:** 10.1007/s10787-019-00648-8

**Published:** 2019-10-08

**Authors:** Xiaoping Zhu

**Affiliations:** Department of Anesthesiology, The First Affiliated Hospital of Nanchang University, 17 Yongwaizheng Street, Nanchang, 330006 China

**Keywords:** Celecoxib, Preemptive analgesia, Femoroacetabular impingement, Hip arthroscopy surgery, Postoperative pain

## Abstract

We aimed to investigate the efficacy of preemptive analgesia of celecoxib on postoperative pain, patients’ global assessment (PGA) and hip function recovery compared to postoperative analgesia of celecoxib in femoroacetabular impingement (FAI) patients who underwent hip arthroscopy surgery (HAS). The 100 FAI patients underwent HAS were randomly allocated to preemptive analgesia group (*N* = 50) or postoperative analgesia group (*N* = 50) as a 1:1 ratio for 3 months. Pain visual analog scale (VAS) score, PGA score, rescue-use pethidine consumption and Harris hip score were assessed. Compared to postoperative analgesia group, pain VAS score decreased on day 1 (*P* = 0.036), day 2 (*P* = 0.046) and day 3 (*P* = 0.046), while was similar prior to operation (*P* = 0.587), on day 7 (*P* = 0.398), at month 1 (*P* = 0.461) and month 3 (*P* = 0.805) in preemptive analgesia group. Besides, rescue-use pethidine consumption was decreased in preemptive analgesia group than postoperative analgesia group within 3 days (*P* = 0.016) and within 7 days (*P* = 0.033) post-operation. For PGA score, it reduced on day 2 (*P* = 0.030) and day 3 (*P* = 0.048), while was similar prior to operation (*P* = 0.699), on day 1 (*P* = 0.699), day 7 (*P* = 0.224), at month 1 (*P* = 0.640) and month 3 (*P* = 0.400) in preemptive analgesia group than postoperative analgesia group. For Harris hip score, it was similar prior to operation (*P* = 0.372), on day 7 (*P* = 0.366), at month 1 (*P* = 0.466) and month 3 (*P* = 0.658) between the two groups. In conclusion, preemptive analgesia of celecoxib decreases short-term postoperative pain and PGA, but without effect on long-term hip function recovery than postoperative analgesia of celecoxib in FAI patients who underwent HAS.

## Introduction

Femoroacetabular impingement (FAI) is a clinical syndrome characterized by the anatomic abnormalities of the femoral head and/or the acetabulum. The anatomic abnormalities result in pathologic contact and shearing forces at the acetabular labrum and cartilage during physiological hip motion (particularly in positions of hip flexion and rotation), thereby giving rise to the damage of cartilage and labrum, as well as hip pain (Amanatullah et al. 2015; Pun et al. 2015). In the last decade, the management of FAI has been evolving rapidly with common use of open hip surgery, periacetabular osteotomy as well as hip arthroscopy surgery (HAS) (Amanatullah et al. 2015; Pun et al. 2015). Among these treatments, HAS represents as an indispensable tool in modern hip-preserving surgery, which has shown obvious promise due to its features including small invasion, fast recovery time and low rate of disability (Gollwitzer et al. 2016). Although great improvement has been achieved with the application of HAS along with the development of imaging technology and surgical instrumentation, postoperative pain remains one of the major causes of FAI patient’s dissatisfaction after HAS during the perioperative period and there still lacks a gold standard for postoperative pain management in this situation (Cogan et al. 2016). Thus, exploring additional and convincing methods for pain management to decrease pain and promote disease recovery in FAI patients underwent HAS is essential.

Celecoxib, a diaryl-substituted pyrazole chemically designated as 4-[5-(4-methylphenyl)-3-(trifluoromethyl)-1*H*-pyrazol-1-yl] benzenesulfonamide, is responsible for prostaglandin synthesis inhibition (that is, an integral part of the pain and inflammation pathway) due to selective inhibition of cyclooxygenase-2 (COX-2), which presents with analgesic effect, anti-inflammatory effect as well as antipyretic activity effect (Cohen and Preuss 2019). A few studies have shown that celecoxib has benefits on reducing pain and preventing heterotopic ossification formation in FAI patients who underwent HAS (Cogan et al. 2016; Kahlenberg et al. 2017). However, whether there are differences between preemptive analgesia and postoperative analgesia of celecoxib in treating FAI patients who underwent HAS is still unclear. Thus, exploration about the optimum dosage time of celecoxib is necessary to improve the pain management and function recovery. Hence, we performed this study and the purpose was to investigate the efficacy of preemptive analgesia of celecoxib on postoperative pain, patients’ global assessment (PGA) and hip function recovery compared to postoperative analgesia in FAI patients who underwent HAS.

## Materials and methods

### Participants

From January 2014 to December 2018, a total of 100 FAI patients who underwent HAS in The First Affiliated Hospital of Nanchang University were consecutively recruited in this study. The patients were eligible if they met the following conditions: (1) diagnosed as FAI according to clinical signs (such as impingement either due to slipped capital femoral epiphysis, acetabular retroversion or coxa profunda, or decreased femoral head-neck offset) and imaging assessment (including radiographs, computed tomography (CT) scan or magnetic resonance imaging (MRI) arthrogram); (2) about to receive HAS; (3) age ≥ 18 years old; (4) no history of hip surgery or interventional therapy. The following patients were excluded: (1) diagnosed as hip osteoarthritis; (2) bilateral lesions; (3) intra-articular treatment with corticosteroid or hyaluronic acid injection in the past 3 months; (4) received analgesic drugs within 7 days before enrollment; (5) hypersensitivity to the study medications (celecoxib or pethidine); (6) hepatic, renal, pancreatic, gastrointestinal, or blood coagulation disorders; (7) pregnancy or lactating woman. This study was approved by the Ethics Committee of The First Affiliated Hospital of Nanchang University in August 2013. All the patients signed informed consents before enrollment.

### Randomization

All eligible patients were randomly allocated to preemptive analgesia group (*N* = 50) or postoperative analgesia group (*N* = 50) as 1:1 ratio. The randomized list was designed by Shanghai Qeejen Institution (Shanghai, China), and the randomization code of patients was generated using blocked randomization method (block length was 6) using SAS 9.0 software (Statistical Analysis System, Cary, USA). When a patient was eligible, an email including patient information was sent to Shanghai Qeejen Institution (Shanghai, China), then a unique subject identification number for the patient was provided by Shanghai Qeejen Institution (Shanghai, China). According to identification numbers, the patients were assigned to corresponding groups and received relevant treatment protocols.

### Treatment

All patients underwent HAS method as described in a previous study (Domb et al. 2012). For preemptive analgesia group, all patients received celecoxib as follows: 400 mg (oral) at 24 h prior to operation, 200 mg (oral) at 12 h and 2 h prior to operation, and then 200 mg (oral) twice a day from 12 h post-operation to day 7 post-operation. For postoperative analgesia group, patients received celecoxib 400 mg (oral) at 12 h post-operation, then 200 mg (oral) twice a day until 7 days post-operation. Within 7 days post-operation, if patients suffered from intolerable pain, they were allowed to receive rescue analgesia with pethidine, and the dosage of used of pethidine was recorded. To prevent heterotopic ossification, all the patients received celecoxib 200 mg (oral) per day from day 8 to month 1 post-operation.

### Data collection

The baseline characteristics including age, gender, side of lesions and FAI type were recorded for each patient. Pain visual analog scale (VAS) score (0–10, 0 indicating no pain, 10 indicating the intolerable pain) and PGA score (0–10, 0 indicating very well condition, 10 indicating worst condition) were evaluated prior to operation, then the postoperative assessments of pain VAS score and PGA score were performed on day 1, day 2, day 3, day 7, at month 1 and month 3, respectively. Harris hip score was assessed before operation, then on day 7 post-operation, at month 1 and month 3 post-operation, respectively. Harris hip score was used to evaluate the function recovery of hip, covering pain (1 item, scored 0–44 points), function (7 items, scored 0–47 points), absence of deformity (1 item, scored 4 points), and range of movement (2 items, scored 5 points), and the maximum Harris hip score was 100 (best possible outcome) (Harris 1969). Besides, the consumption of rescue-use pethidine within 3 days operation and within 7 days post-operation was also recorded, respectively.

### Sample size calculation

To calculate the required sample size, we assumed that the mean pain VAS score and standard deviation (SD) on day 3 post-operation was 5.0 ± 1.5 in preemptive analgesia group and 6.0 ± 1.5 in postoperative analgesia group. Based on the assumed pain VAS score of each group, the required sample size was 42 in each group using a two-sided two-sample *t* test, 5% level of significance (α) and a power of 0.85, which was calculated by PASS V11.0 software (NCSS, Kaysville, UT, USA). While considering that there was at least 10% attrition rate, the sample size was finally increased to 50 in each group.

### Statistical analysis

Based on intention-to-treat (ITT) principle, all patients were included in the final analysis. As for patients who lost follow up or withdrew from the study, the data at the last follow-up were used as the values of each later missing visit. Continuous variables were presented as mean and standard deviation (SD), categorical variables were displayed as count (percentage). Comparisons between groups were determined by Student’s *t* test or Chi-square test. Data analyses were performed using SPSS 24.0 (IBM, Chicago, USA), and figures were plotted using GraphPad Prism 7.00 (GraphPad Software, San Diego, USA). All tests were two-sided, and *P* value < 0.05 was considered as significant.

## Results

### Study flow

**Fig. 1:**
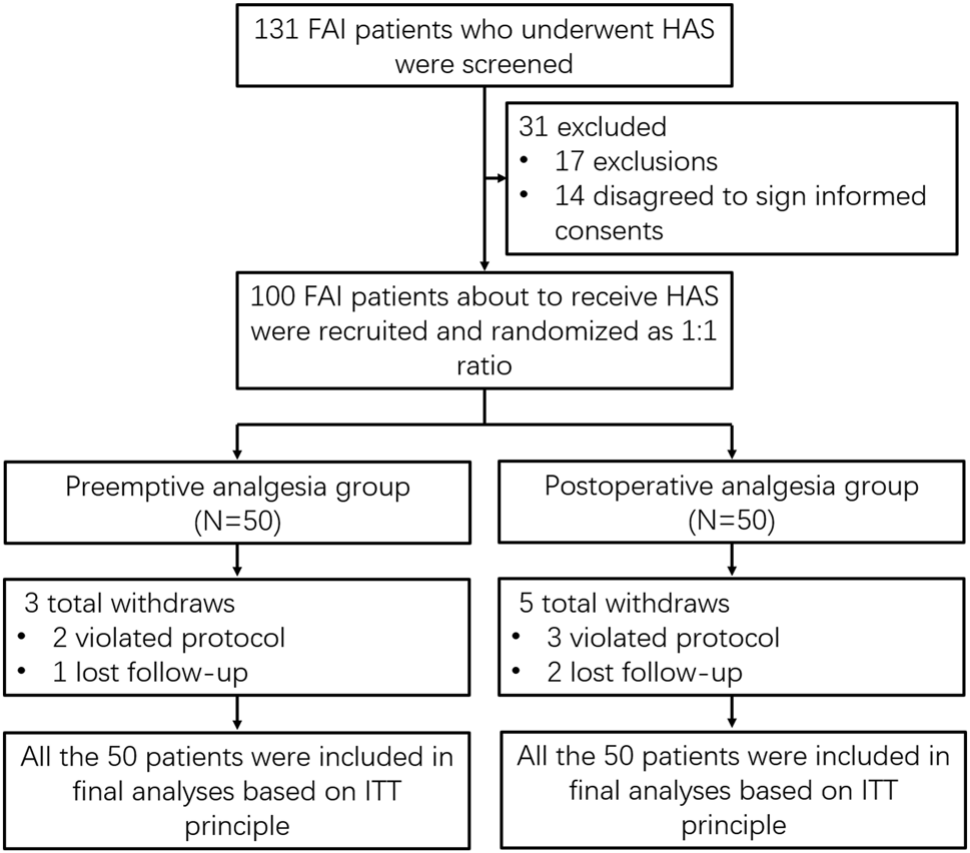
Study flow. *FAI* femoroacetabular impingement, *HAS* hip arthroscopy surgery, *ITT* intention-to-treat

A total of 131 FAI patients who underwent HAS were screened, while 31 patients were excluded (including 17 patients who did not meet the inclusion criteria or met the exclusion criteria, and 14 patients who disagreed to sign the informed consents) (Fig. 1). The remaining 100 patients who were about to receive HAS were recruited and randomly allocated to preemptive analgesia group (*N* = 50) or postoperative analgesia group (*N* = 50) as 1:1 ratio. In the preemptive analgesia group, there were three patients who withdrew (including two patients who violated protocol and one patient who lost follow-up), whereas in the postoperative analgesia group, there were five patients who withdrew (including three patients who violated protocol and two patients who lost follow-up). All 50 patients in preemptive analgesia group and 50 patients in postoperative analgesia group were included in final analyses based on ITT principle.

### FAI patients’ characteristics

No difference was found in age (*P* = 0.370) or gender (*P* = 0.548) between preemptive analgesia group and postoperative analgesia group (Table 1). The mean age of FAI patients in preemptive analgesia group and postoperative analgesia group was 34.3 ± 6.2 years and 35.5 ± 7.1 years, respectively. And there were 27 (54.0%) males as well as 23 (46.0%) females in preemptive analgesia group, and 24 (48.0%) males as well as 26 (52.0%) females in postoperative analgesia group. Based on FAI type, the numbers of patients with cam type, pincer type and mixed type were 24 (48.0%), 16 (32.0%) and 10 (20.0%), respectively, in preemptive analgesia group, and were 22 (44.0%), 19 (38.0%) and 9 (18.0%), respectively, in postoperative analgesia group. Furthermore, the mean value of pain VAS score, PGA score and Harris hip score were 6.4 ± 1.5, 7.3 ± 1.7 and 58.2 ± 7.5, respectively, in preemptive analgesia group, and were 6.5 ± 1.6, 7.5 ± 1.8 and 56.8 ± 8.1, respectively, in postoperative analgesia group. There was no difference in FAI type (*P* = 0.673), pain VAS score (*P* = 0.587), PGA score (*P* = 0.674) or Harris hip score (*P* = 0.372) between the two groups.

**Table 1:** Characteristics of FAI patients

Parameters	Preemptive analgesia group (*N* = 50)	Postoperative analgesia group (*N* = 50)	*P* value
Age (years), mean ± SD	34.3 ± 6.2	35.5 ± 7.1	0.370
Gender, No (%)			0.548
Male	27 (54.0)	24 (48.0)	
Female	23 (46.0)	26 (52.0)	
Side, No (%)			0.688
Left	28 (56.0)	26 (52.0)	
Right	22 (44.0)	24 (48.0)	
FAI type, No (%)			0.673
Cam	24 (48.0)	22 (44.0)	
Pincer	16 (32.0)	19 (38.0)	
Mixed	10 (20.0)	9 (18.0)	
Pain VAS score, mean ± SD	6.4 ± 1.5	6.5 ± 1.6	0.587
PGA score, mean ± SD	7.3 ± 1.7	7.5 ± 1.8	0.674
Harris hip score, mean ± SD	58.2 ± 7.5	56.8 ± 8.1	0.372

Comparison was determined by Student’s *t* test or Chi-square test

*FAI* femoroacetabular impingement, *SD* standard deviation, *VAS* visual analogue scale, *PGlA* patient’s global assessment

### Comparison of pain VAS score

**Fig. 2:**
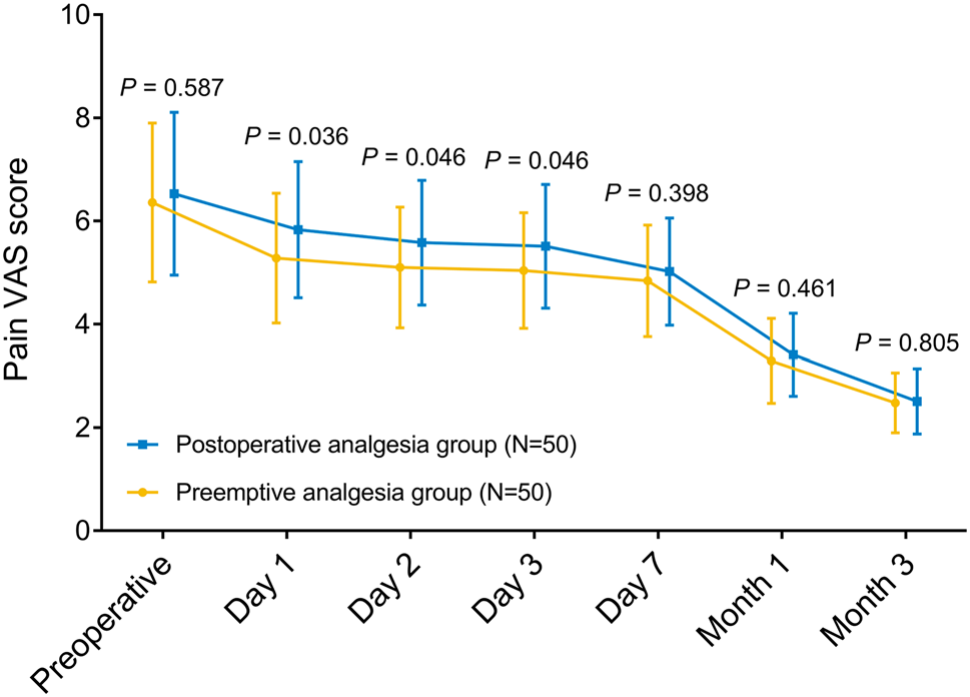
Pain VAS score. Comparisons of pain VAS score between preemptive analgesia group and postoperative analgesia group prior to operation, on day 1, day 2, day 3 as well as day 7, and at month 1 as well as month 3 were determined by Student’s *t* test. *VAS* visual analog scale

Pain VAS score was decreased in preemptive analgesia group compared to postoperative analgesia group on day 1 (*P* = 0.036), day 2 (*P* = 0.046) and day 3 (*P* = 0.046), while it was similar prior to operation (*P* = 0.587), on day 7 (*P* = 0.398), at month 1 (*P* = 0.461) as well as month 3 (*P* = 0.805) between the two groups (Fig. 2).

### Comparison of PGA score

**Fig. 3:**
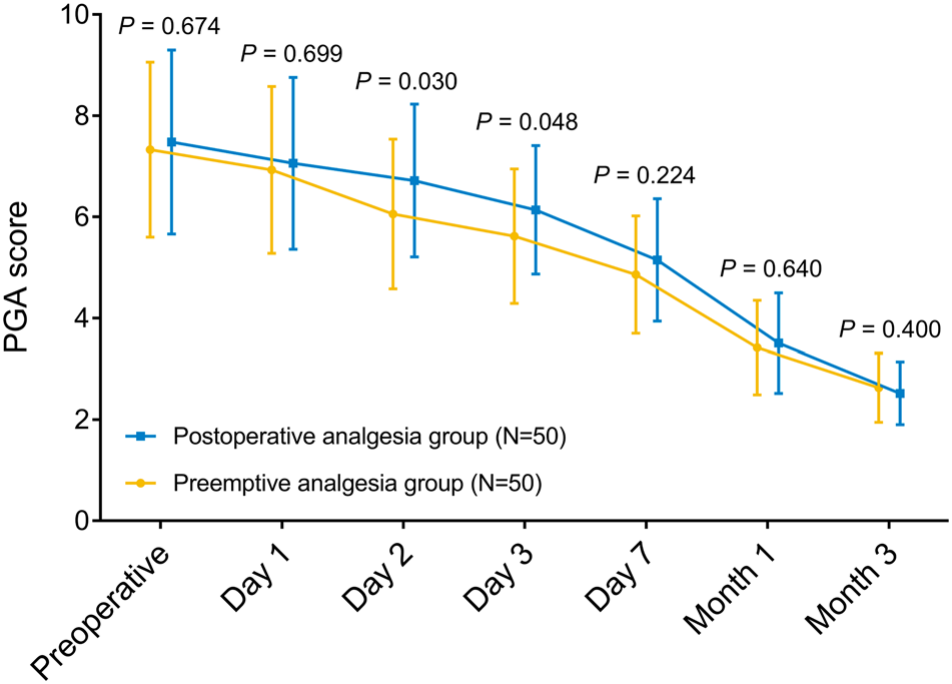
PGA score. Comparisons of PGA score between preemptive analgesia group and postoperative analgesia group prior to operation, on day 1, day 2, day 3 as well as day 7, and at month 1 as well as month 3 were determined by Student’s *t* test. *PGA* patient’s global assessment

PGA score was reduced in preemptive analgesia group compared to postoperative analgesia group on day 2 (*P* = 0.030) and day 3 (*P* = 0.048), whereas it appeared to be of no difference prior to operation (*P* = 0.699), on day 1 (*P* = 0.699), day 7 (*P* = 0.224), at month 1 (*P* = 0.640) and month 3 (*P* = 0.400) between the two groups (Fig. 3).

#### Comparison of the consumption of rescue-use pethidine

**Fig. 4:**
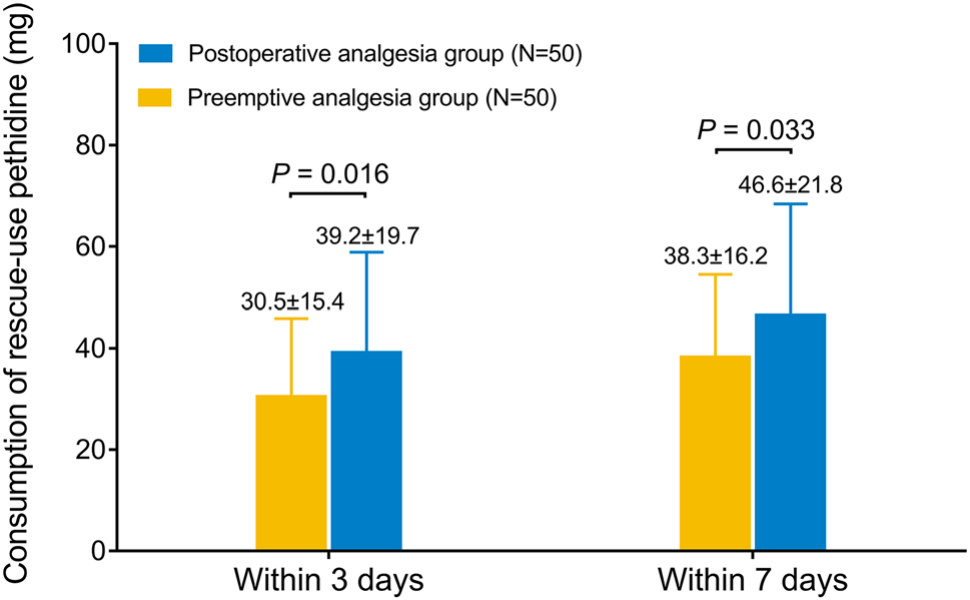
The consumption of rescue-use pethidine. Comparisons of consumption of rescue-use pethidine between preemptive analgesia group and postoperative analgesia group within 3 days post-operation and within 7 days post-operation were determined by Student’s *t* test

The consumption of rescue-use pethidine was decreased in preemptive analgesia group compared with postoperative analgesia group within 3 days post-operation (mean value 30.5 ± 15.4 vs. 39.2 ± 19.7) (*P* = 0.016), as well as within 7 days post-operation (mean value 38.3 ± 16.2 vs. 46.6 ± 21.8) (*P* = 0.033) (Fig. 4).

### Comparison of Harris hip score

**Fig. 5:**
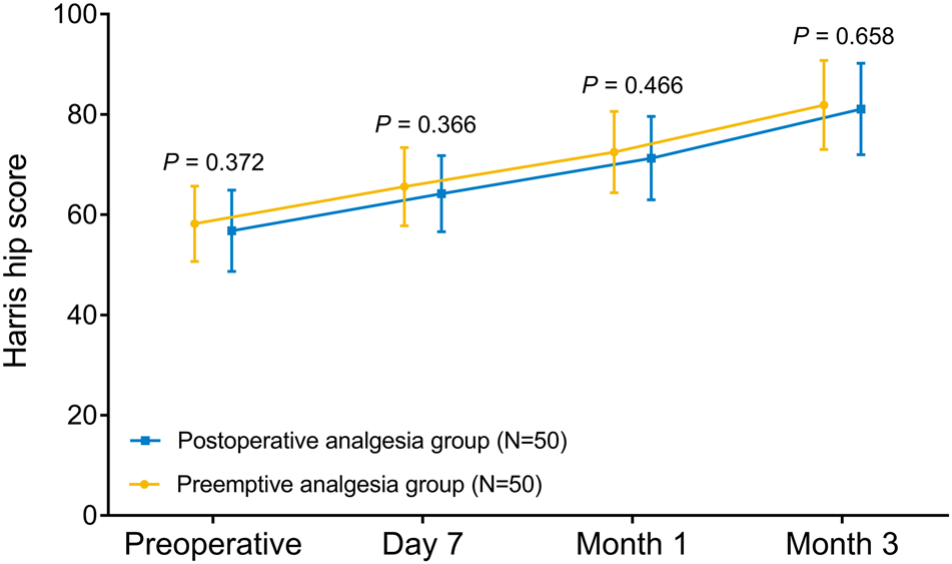
Harris hip score. Comparisons of Harris hip score between preemptive analgesia group and postoperative analgesia group prior to operation, on day 7, at month 1 and at month 3 were determined by Student’s *t* test

No difference was discovered in Harris hip score between preemptive analgesia group and postoperative analgesia group prior to operation (*P* = 0.372), on day 7 (*P* = 0.366), at month 1 (*P* = 0.466) and at month 3 (*P* = 0.658) (Fig. 5).

## Discussion

Despite the advancement of HAS technique in the last decade, perioperative pain management remains a considerable challenge. Increased pain and anaesthetic consumption might directly decrease patient’s satisfaction, reduce the quality of recovery as well as prolong discharge time (Shin et al. 2018). Regarding to this, perioperative pain management with efforts to control pain in the acute postoperative period is needed. Celecoxib, a type of non-steroidal antiinflammatory drug (NSAID), presents with the distinct characteristics including rapid absorption, preferential distribution into inflamed tissue as well as high oral bioavailability, which has been studied in the setting of HAS and been reported to be effective in reducing pain and decreasing anaesthetic consumption in FAI patients (Holmes et al. 2013; Kahlenberg et al. 2017). One randomized, double-blinded controlled study in FAI patients who underwent HAS reveals that celecoxib-treated patients have decreased pain (during the acute postoperative period after HAS) and reduced discharge time compared to the placebo-treated patients (Kahlenberg et al. 2017). Similarly, another randomized placebo-controlled study in FAI patients who underwent HAS also discloses that celecoxib-treated patients present with improved pain control and accelerated recovery compared to the placebo-treated patients (Zhang et al. 2014).

Although these previous studies have confirmed the good efficacy of celecoxib in pain management after HAS in FAI patients, the information about the optimum time to use celecoxib (preemptive or postoperative) for pain management in FAI patients underwent HAS is still unclear. Therefore, investigating the optimum time for the use of celecoxib is necessary, which might have critical effects on improving postoperative pain management for FAI. We carried out this randomized controlled study to compare the efficacy of preemptive analgesia of celecoxib versus its postoperative use on pain management and PGA in FAI patients who underwent HAS, and we discovered that preemptive analgesia of celecoxib presented with better efficacy on decreasing short-term pain VAS score and short-term PGA score compared to postoperative analgesia of celecoxib in FAI patients who underwent HAS. The possible explanations were as follows: celecoxib, a type of NSAID with distinct properties of inhabitation of COX-2 isoenzyme, could promote its preferential distribution into inflamed tissue as well as possess good oral bioavailability, which was a good option for perioperative pain management and reduced consumption of pethidine (Cohen and Preuss 2019). Considering its plasma peak concentration reached about 2–4 h after oral intake, and its half-life was 8–12 h, preemptive analgesia of celecoxib could play its therapeutic effect and remain the peak drug effects during the procedures of HAS. Also it reach the steady-state concentration as soon as possible to reposefully provide analgesia effect, which directly increased short-term pain control and decreased short-term PGA score in FAI patients who underwent HAS (Davies et al. 2000).

Conventional pain management after several orthopedic operations depends heavily on the application of anaesthetic medications (including pethidine), whereas these anaesthetic medications lead to several postoperative side effects on various systems (including gastrointestinal, respiratory, neurologic as well as cardiovascular systems) and poor postoperative quality of recovery (Horlocker 2010). According to previous studies, celecoxib exhibits good effects on decreasing anaesthetic medication consumption in FAI patients who underwent HAS, while, whether preemptive analgesia of celecoxib has better efficacy on reducing anaesthetic consumption compared to postoperative analgesia is still largely unknown in FAI patients who underwent HAS. In the present study, we also recorded the consumption of rescue-use pethidine within 3 days and within 7 days post-operation, and found that preemptive analgesia of celecoxib displayed better effects on reducing the consumption of rescue-use pethidine compared to postoperative analgesia in FAI patients who underwent HAS, which might be caused by that celecoxib (used before opeartion) presented with good efficacy on short-term pain control owning to its good oral bioavailability and short half-life (above-mentioned), subsequently it might have reached a steady-state concentration to reposefully provide analgesia effect, thereby decreased the frequency of pain outbreak and reduced consumption of rescue-use pethidine in FAI patients who underwent HAS.

For the sake of investigating whether there were differences in the long-term pain management and hip function recovery between preemptive analgesia and postoperative analgesia of celecoxib in FAI patients underwent HAS, we also assessed the pain management and disease recovery at month 1 as well as month 3 of FAI patients, and we found no difference in the efficacy of celecoxib on decreasing pain VAS score and PGA score in long duration in FAI patients who underwent HAS. The possible reasons were that celecoxib would reach the steady-state concentration and provide a consistent effect on pain controls after used in both preemptive analgesia and postoperative analgesia. Thus, there was no influence on its efficacy for long-term pain management and PGA between preemptive analgesia and postoperative analgesia in FAI patients underwent HAS. In addition, we also discovered that Harris hip score was similar between preemptive analgesia of celecoxib and postoperative analgesia of celecoxib in FAI patients who underwent HAS. The possible explanations were that the hip function recovery might heavily rely on its disease severity, operation conditions, long-term rehabilitation training and physical exercises, thus, there might be no obvious effect of celecoxib on hip function recovery in FAI patients who underwent HAS (Kierkegaard et al. 2017).

Interesting results were discovered in the present study, while several limitations still existed. (1) This study was a single-center study and the sample size of 100 FAI patients who underwent HAS was relatively small, which might cause relatively poor statistical power. Thus, further study enrolling more patients from multicenters was greatly needed. (2) The difference in operation experience of disparate doctors might cause deviation. (3) Due to the difficulty of long-term follow-up, we only evaluated the efficacy of preemptive analgesia of celecoxib on postoperative pain management and hip function recovery compared to postoperative analgesia in FAI patients who underwent HAS within 3 months, while further study exploring its longer term effect is great needed.

## Conclusions

In conclusion, preemptive analgesia of celecoxib decreases short-term postoperative pain and PGA, while has little effect on long-term hip function recovery compared to postoperative analgesia of celecoxib in FAI patients underwent HAS. These findings provide additional evidence for the optimum time of celecoxib use, which might be beneficial for rapid disease recovery in FAI patients who underwent HAS.
